# Supplementary material for: Modification of Tomato Photosystem II Photochemistry with Engineered Zinc Oxide Nanorods
Source: Plants (Basel). 2023 Oct 8;12(19):3502. doi: 10.3390/plants12193502 (PMC10575289; doi:10.3390/plants12193502)
Supplement: Supplementary file 1 [file plants-12-03502-s001.zip › plants-2650753-supplementary.pdf]

plants

Modification of Tomato Photosystem II Photochemistry with engineered Zinc Oxide Nanorods

Panagiota Tryfon, Ilektra Sperdouli, Ioannis-Dimosthenis S. Adamakis, Stefanos Mourdikoudis, Catherine Dendrinou-Samara and Michael Moustakas

Table S1. Definitions of the chlorophyll fluorescence parameters used in the experiments.

Parameter	Definition	Calculation
F*v*/F*m*	Maximum efficiency of PSII photochemistry	(F*m* − F*o*)/F*m*
Φ_{PSII}	Effective quantum yield of PSII photochemistry	(F*m*′ − F*s*)/F*m*′
Φ_{NPQ}	Quantum yield of regulated non-photochemical energy loss in PSII	F*s*/F*m*′ − F*s*/F*m*
Φ_{NO}	Quantum yield of nonregulated energy loss in PSII	F*s*/F*m*
F*v*′/F*m*′	Efficiency of open PSII reaction centers	(F*m*′ – F*o*′)/F*m*′
F*v*/F*o*	Efficiency of the oxygen evolving complex (OEC) on the donor side of PSII	(F*m* − F*o*)/F*o*
ETR	Electron transport rate	Φ_{PSII} × PAR × c × abs, where PAR is the photosynthetically active radiation, c is 0.5, and abs is the total light absorption of the leaf taken as 0.84
q*p*	Photochemical quenching, representing the redox state of quinone A (Q_A), or in other words the fraction of PSII reaction centers in open state	(F*m*′ – F*s*)/(F*m*′ – F*o*′)
NPQ	Non-photochemical quenching reflecting the dissipation of excitation energy as heat	(F*m* – F*m*′)/F*m*′
EXC	Excess excitation energy	(F*v*/F*m* – Φ_{PSII})/F*v*/F*m*

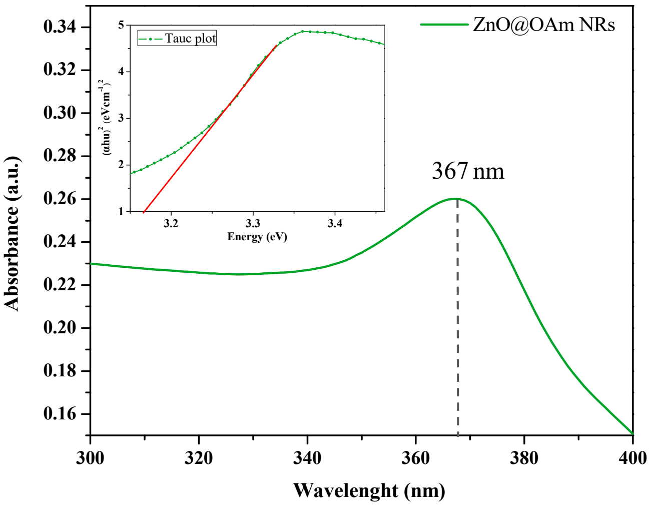

Figure S1. UV-Vis spectrum and optical band gap energy plot (inset) of ZnO@OAm NRs.

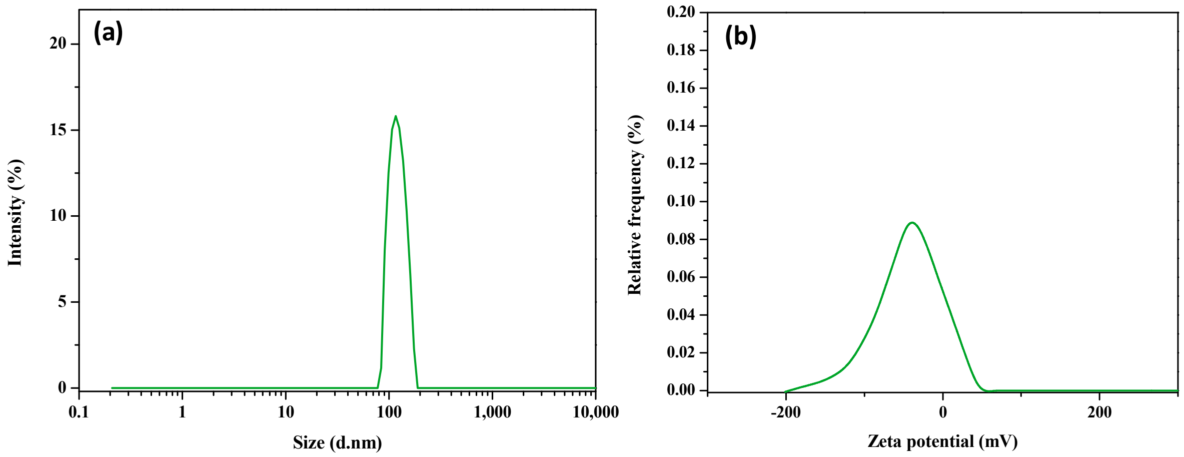

Figure S2. Dynamic light scattering (DLS) spectra, hydrodynamic size (122 nm) (**a**) and the ζ-potential (-4.8 mV) of NRs (**b**). Analyses performed in ethanol/water solution (1:3).
